# Significance of initial, interim and end-of-therapy ^18^F-FDG PET/CT for predicting transformation risk in follicular lymphoma

**DOI:** 10.1186/s12935-021-02094-5

**Published:** 2021-07-26

**Authors:** Mixue Xie, Lulu Wang, Qi Jiang, Xuxia Luo, Xin Zhao, Xueying Li, Jie Jin, Xiujin Ye, Kui Zhao

**Affiliations:** 1 Department of Haematology, The First Affiliated Hospital, College of Medicine, Zhejiang University, Hangzhou, 310003 Zhejiang China; 2 Department of Nuclear Medicine, The First Affiliated Hospital, College of Medicine, Zhejiang University, Hangzhou, 310003 Zhejiang China; 3 Department of Medical Oncology, The First Affiliated Hospital, College of Medicine, Zhejiang University, Hangzhou, 310003 Zhejiang China

**Keywords:** Follicular lymphoma, Histological transformation, Maximum standardized uptake value, 18F-FDG PET/CT, Predictive significance

## Abstract

**Background:**

Histological transformation (HT) of follicular lymphoma to a more aggressive lymphoma is a serious event affecting patients’ outcomes. To date, no strong clinical HT predictors present at diagnosis have yet been identified. The fluorodeoxyglucose (FDG)-positron emission tomography (PET)/computed tomography (CT) is highlighted as a non-invasive diagnostic tool for the detection of HT, but its ability to predict HT at early stage of disease has not been clear. Therefore, this study investigated the predictive values of the pre-transformation standardized uptake value (SUV_max_) for the risk of transformation in FL.

**Methods:**

This retrospective study involved 219 patients with FL between June 2008 and October 2019 who had undergone ^18^F-FDG PET/CT scan. One hundred and thirty-two, 64, and 78 patients underwent PET at baseline (PET_baseline_), interim (PET_interim_) and end-of-induction therapy (PET_end_), respectively. Qualitative assessment was performed using the 5-point Deauville scale. Statistical analysis was done using Cox regression models, receiver operating characteristic (ROC) analysis, and Kaplan–Meir survival curves.

**Results:**

Of the 219 patients included, 128 had low-grade FL (grade 1–2) and 91 had high-grade FL (grade 3a). HT eventually occurred in 30 patients. The median time to HT was 13.6 months. Among clinical indicators, advance pathological grade was shown as the most significant predictor of HT (HR = 4.561, 95% CI 1.604–12.965). We further assessed the relationship between PET and HT risk in FL. Univariate Cox regression determined that SUV_baseline_ and SUV_end_ were significant predictors for HT, while neither SUV_interim_ nor qualitative assessment of Deauville score has predictive value for HT. Due to the noticeable impact of high pathological grade on the HT risk, we conducted the subgroup analysis in patients with low/high pathological grade, and found SUV_baseline_ could still predict HT risk in both low-grade and high-grade subgroups. Multivariate analysis adjusted by FLIPI2 score showed the SUV_baseline_ (HR 1.065, 95% CI 1.020–1.111) and SUV_end_ (HR 1.261, 95% CI 1.076–1.478) remained as significant predictors independently of the FLIPI2 score. According to the cut-off determined from the ROC analysis, increased SUV_baseline_ with a cutoff value of 14.3 and higher SUV_end_ with a cutoff value of 7.3 were highly predictive of a shorter time to HT.

**Conclusions:**

In follicular lymphoma, quantitative assessment used SUV_max_ at the pre-treatment and end-of-treatment PET/CT scan may be helpful for early screen out patients at high risk of transformation and guide treatment decisions.

**Supplementary Information:**

The online version contains supplementary material available at 10.1186/s12935-021-02094-5.

## Background

Follicular lymphoma (FL) is the most common form of inert non-Hodgkin's lymphoma (NHL), accounting for 7–15% of all lymphomas worldwide [1]. The clinical course of FL is highly heterogeneous. Although many patients exhibit the characteristics of indolent lymphoma and can survive for a long time, others show rapid progression and often transformation to a more aggressive form of lymphoma, usually diffuse large B-cell lymphoma (DLBCL), with an annual incidence of 2–3% [2]. Transformation is associated with increased morbidity and mortality. Although the development of rituximab has greatly improved the prognosis of such patients, the survival of patients with HT is still not optimistic, with 5-year OS and PFS reported to be 68% and 58% for patients with HT at primary diagnosis, and even worse with 5-year OS and PFS rates of only 68% and 18% for patients with sequentially HT [3]. Therefore, there is a need to identify predictive markers of HT risk to guide in clinical decision-making so that high-risk patients can benefit from more aggressive treatment. Currently, the prognostic assessment for FL is usually based on the FL International Prognostic Index 2 (FLIPI2), which has been the strongest predictor of FL prognosis but weaker in predictive ability for HT risk [3]. So far, there are no strong clinical HT predictors present at the time of first diagnosis identified [3–6]. The initial treatment strategy watch-and-wait approach and higher pathological grades appear to more significantly affect FL transformation [7].

Over the past decade, ^18^F-FDG PET/CT has been widely used in the clinic of lymphoma, including disease diagnosis, initial staging, and follow-up after treatment [8, 9]. The maximum standardized uptake value (SUV_max_) derived from PET/CT is the most commonly used quantitative index as a standard of malignancy, and a higher value is generally considered a poor prognostic factor [10]. The First International Workshop held in Deauville, France in 2009 proposed new PET/CT assessment standard for lymphoma named the Deauville standard comprising a 5-point scale (D5PS), which defined a simple visual response criteria at interim and end-of-treatment [11]. In recent years, the quantitative and qualitative indicators of PET/CT have been studied for predicting the prognosis of FL, and the results were inconclusive and contradictory. Most studies suggested higher values of quantitative SUV_max_ and total metabolic tumor volume (TMTV) parameters, and qualitative Deauville index are considered to be associated with inferior survival [12–14], while the other study unexpectedly reported that a low SUV_max_ correlated with a worse PFS [15]. In term of HT in FL, PET/CT is commonly used as a non-invasive diagnostic tool for the detection of HT and to determine the best suitable re-biopsy site in the context of relapse [16]. There are very few studies evaluating the ability of PET/CT metrics to predict the risk of HT at early diagnosis stage. Mir et al. assessed the relationship between baseline SUV_max_ and the risk for HT in FL from the GALLIUM study, and found no temporal relationship between baseline SUV_max_ and HT; neither baseline SUV_max_ nor baseline SUV_range_ could predict HT [17]. However, in the study all included patients had high tumor burden, and only 15 of them were patients with HT. The limited sample size and single patient population may lead to bias in the results. More studies are needed to determine the predictive value PET/CT-based measurements for HT risk both in low-grade/tumor burden or high-grade/tumor burden patients. Moreover, the usefulness of interim and terminal PET/CT assessments in predicting HT has not been reported to date, so the significance of PET/CT parameters at different treatment time points remains to be further studied.

To this end, in this study, we investigated the correlation between clinical indicators and HT risk, and further analyzed the quantitative SUV_max_ parameter and qualitative D5PS index of PET/CT evaluation at different treatment stages in predicting HT risk. Subgroup analysis was conducted in patients with low and high pathological grade, and the best cutoff values of PET/CT parameters were calculated as the basis for early screening and judgment of high-risk HT patients.

## Patients and methods

### Patients

Two hundred and twenty-eight patients with histologically proven FL underwent PET/CT from June 2008 to October 2019. Among them, 219 patients who met the following inclusion criteria were included: patients having histologically confirmed FL with grading, including subsequent histologically confirmed transformation to other invasive lymphomas; patients having no evidence of biopsy-confirm transformation when underwent PET/CT scan; patients aged ≥ 18 years; and patients whose PET_baseline_, PET_interim_ (after 3 or 4 cycles of chemotherapy), or PET_end_ (after 5 or 6 cycles of chemotherapy) data were available. The treatment of low-grade (1–2) FL was decided by the hematooncologist depending on the patient’s condition. High-grade FL (3a) was treated with the following regimens: R-CHOP (rituximab, cyclophosphamide, doxorubicin, vincristine, and prednisone) and R-CVP (rituximab, cyclophosphamide, vincristine and prednisone). Nine individuals were excluded: two for whom pathologic grading data were lacking, two who showed lower FDG uptake in the tumor than in the liver at PET/CT_baseline_, four who received radiotherapy due to a massive mass but did not receive chemotherapy, and one for whom time data for transformation were lacking. Clinical parameters and hematological data (Ann Arbor stage, NCCN-FLIPI1, NCCN-FLIPI2, pathological grade, lactate dehydrogenase [LDH] level, bone marrow involvement, area of enlarged lymph nodes, hemoglobin (HGB) level, fibrinogen, and subsequent treatment planned) were obtained from the patients’ medical records. The study was conducted per the ethical principles of the Declaration of Helsinki. All patients provided written informed consent to participate in the study (Additional file 1).

### PET/CT technique and evaluation

The patients received an intravenous injection of ^18^F-FDG at 4.44–5.55 MBq/kg of body weight after at least 4–6 h of fasting. Adequate hydration was ensured by giving the patients 1000 ml of water 1 h before image acquisition. Blood glucose levels were checked in all patients before FDG injection. The patients’ blood glucose levels were lower than 11 mmol/L. Whole-body PET/CT scans were acquired using a combined PET/CT scanner (Siemens Biograph Sensation 16, Germany). The whole-body CT and PET covered a region ranging from the meatus of the ear to the mid-thigh. PET/CT was performed approximately 1 h after FDG injection. The procedure for data acquisition was as follows: 16-section multi-detection row CT scanning was performed first, from the head to the mid-thigh with 120 kV and 100 mA. The tube rotation time and section thickness were 0.5 s and 5 mm, respectively, which matched the PET section thickness. Immediately after the CT scan, a whole-body PET scan was performed with 1.5- to 2-min acquisition per bed position by using a 3-dimensional acquisition mode. Attenuation-corrected PET images were reconstructed with an ordered-subset expectation maximization iterative reconstruction algorithm (8 subsets, 3 iterations). PET, CT, and fused PET/CT images were generated and reviewed on a Syngo workstation. The scans were independently interpreted by two staff members of the PET center. Disagreements between respective independent interpretations were resolved by consensus.

The SUV refers to the ratio of the radioactivity of the imaging agent taken from local tissues to the average injection activity of the whole body. SUV_max_ was obtained by a software program from Siemens. The PET_Interim_ and PET_end_ scans were assessed using the D5PS as follows: 1, no intake; 2, intake less than or equal to that in the mediastinum; 3, intake higher than that in the mediastinum but less than that in the liver; 4, intake moderately higher than that in the liver; and 5, intake significantly higher than that in the liver or at a new disease site.

### Statistical analysis

**Fig. 1:**
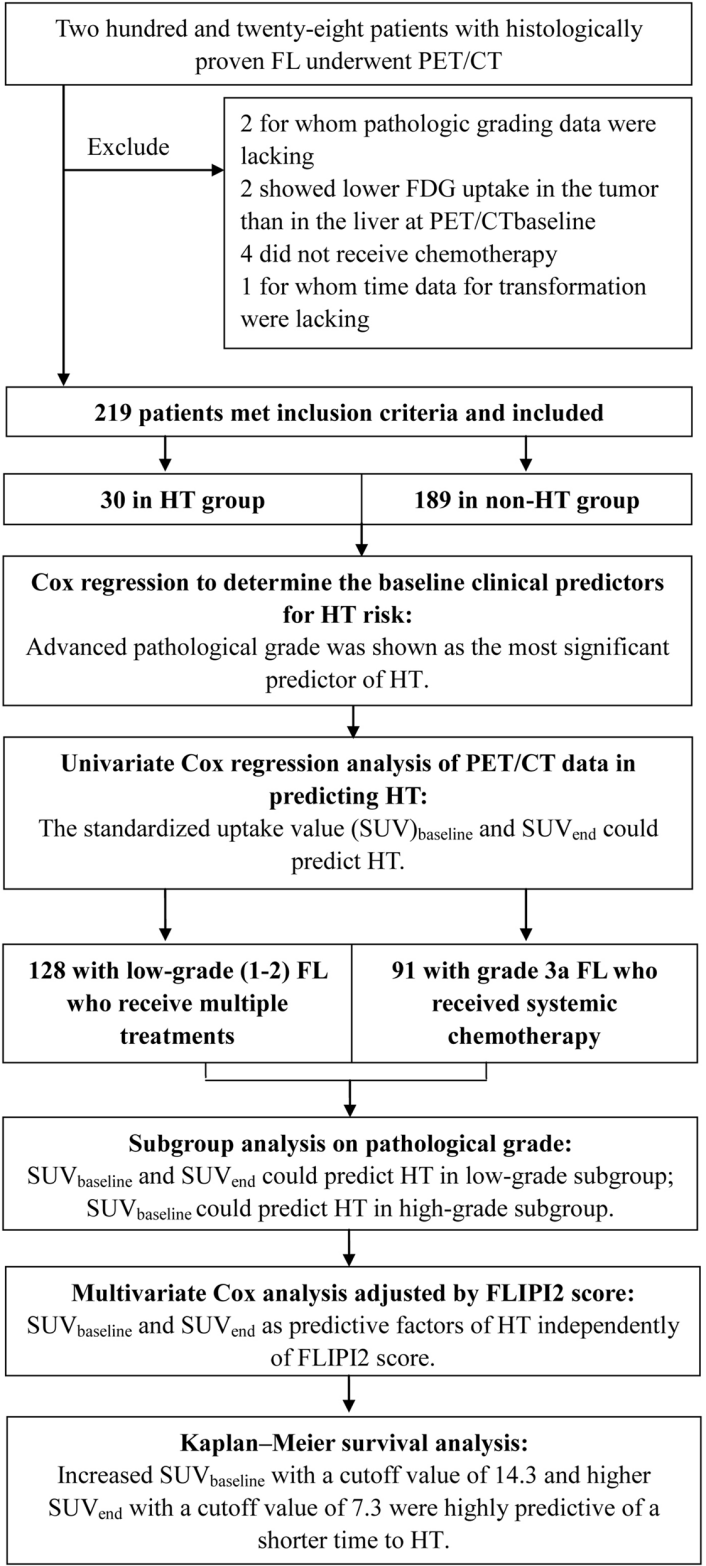
A flowchart of the study protocol and methodology

Continuous variables were expressed as mean ± SD values. Estimates of the predictive effect for HT were expressed as hazard ratios (HRs) in the Cox proportional hazard regression analysis with 95% confidence intervals (CI). Receiver operating characteristic (ROC) analysis was performed to determine the optimal cut-off values for PET parameters for predicting HT. Transformation-free survival curves were constructed using the Kaplan–Meier method and were compared with the log-rank test. Differences between the results of comparative tests were considered significant if the 2-sided P-value was < 0.05. All statistical analyses were performed using STATA version 12.0 (StataCorp, College Station, TX, USA). The flowchart of the study protocol and methodology was shown in Fig. 1.

## Result

### Patient characteristics

Of the 219 patients included (median follow-up: 46 months), 128 had low-grade FL (grade 1–2) and 91 had high-grade (grade 3a) FL. Thirty (13.7%) patients showed biopsy-confirmed HT to diffuse large B-cell lymphoma (DLBCL), with six cases (4.7%) categorized in the low-grade (1–2) group and 24 (26.4%) in the high-grade (3a) group. The median time to transformation was 13.6 months, and the earliest transformation was reported at 186 days (6 months). In the low-grade group, treatment was determined according to the Groupe d’Etude des Lymphomes Folliculaires (GELF) criteria for tumor load (lymph node involvement, large mass, symptom B, splenomegaly, pleural effusion, and blood abnormalities). Fifty-one (39.8%) patients adopted a watch-and-wait approach, 47 (36.7%) received involved site radiation therapy (ISRT) for a local mass, and only 30 (23.4%) received systemic chemotherapy. In the high-grade group, all included patients received chemotherapy, with 61 (67%) receiving R-CHOP and 30 (33%) receiving R-CVP. The median patient age was 51.1 years, and 47% of the patients were male. Table 1 shows baseline characteristics of patients with HT and those that did not show transformation. In comparison with the non-transformed patients, HT patients were older, had higher LDH and fibrinogen levels, and showed a higher proportion of high-grade FL.

**Table 1:** Clinical characteristics and parameters of patients with or without HT

	**HT**	**Non-HT**	***P*-value**
Total number of patients	30	189	
Age, years (range)	50.8 ± 6.2 (26–86)	51.2 ± 14.0 (22–71)	0.047
ECOG (0–1/ ≥ 2)	28/2	184/5	0.149
LDH, U/L (mean ± SD)	279 ± 122	231 ± 119	0.136
Bone marrow involvement	11(36.6%)	67(35.4%)	0.510
Area of enlarged lymph nodes (≥ 4)	16(53.3%)	108(52.9%)	0.601
HGB, g/L (mean ± SD)	132.5 ± 21.2	135.4 ± 18.0	0.470
Fibrinogen, g/L (mean ± SD)	3.54 ± 1.23	2.68 ± 0.98	0.001
FLIPI			0.165
Low (0–1)	5 (9%)	43 (26.7%)	
Intermediate (2)	8 (36.3%)	64 (39.7%)	
High (≥ 3)	9 (40.9%)	54 (33.5%)	
FLIPI2			0.059
Low (0–1)	13 (56.5%)	105 (65.2%)	
Intermediate (2)	6 (26%)	39 (24.2%)	
High (≥ 3)	4 (17.4%)	17 (10.5%)	
Pathologic grade			0.000
Low (1–2)	24 (80%)	67 (35.4%)	
High (3a)	6 (20%)	122 (64.5%)	

### Association of baseline characteristics and the risk of HT

Univariate Cox regression was used to determine the baseline characteristics that could serve as predictors for HT. The results showed that older age, higher fibrinogen levels, higher FLIPI2 score, and advanced pathological grade were correlated with transformation (Table 2). Subsequently, multivariate Cox regression was used for the four significant variables identified by univariate analysis. Higher fibrinogen level (hazard ratio [HR] = 1.385, 95% confidence interval [CI] 1.002–1.915; P = 0.048), higher FLIPI2 score (HR = 1.550, 95% CI 1.002–2.398; P = 0.049), and advanced pathological grade (HR = 4.561, 95% CI 1.604–12.965; P = 0.004) were significant independent predictors of HT. Increased fibrinogen level and higher FLIPI2 score showed modest HR values, ranging from 1.3 to 1.6. Advanced pathological grade was highly predictive of HT risk with HR value of > 4.

**Table 2:** The Cox proportional hazard regression of clinical parameters for HT

	Univariate Cox regression			Multivariate Cox regression		
	**HR**	95%CI	*P*-value	HR	95%CI	*P*-value
Age, year	1.037	1.005–1.071	0.024	1.002	0.964–1.040	0.921
ECOG	1.720	0.940–3.148	0.079			
LDH, U/L	1.001	0.999–1.003	0.080			
Bone marrow involvement	1.304	0.604–2.813	0.499			
Area of enlarged lymph nodes (≥ 4)	0.843	0.405–1.754	0.648			
HGB, g/L	0.992	0.973–1.013	0.491			
Fibrinogen, g/L	1.612	1.206–2.154	0.001	1.385	1.002–1.915	0.048
FLIPI	1.253	0.878–1.788	0.212			
FLIPI2	1.557	1.058–2.291	0.025	1.550	1.002–2.398	0.049
Pathologic grade (grade 3a)	6.034	2.466–14.766	0.000	4.561	1.604–12.96	0.004

### Predictive value of PET/CT data for HT risk in FL

Among the 219 patients with PET/CT data, 132 underwent pre-treatment PET/CT evaluation (PET_baseline_), 64 received post-treatment interim evaluation (PET_interim_, after 3 or 4 cycles), and 78 underwent post-treatment terminal evaluation (PET_end_, after 5 or 6 cycles). The median SUV_baseline_ was 8.1 (range, 0.7–46.6), median SUV_interim_ was 2.9 (range, 0–17.3), and median SUV_end_ was 2.6 (range, 0–20.2). SUV_interim_ and SUV_end_ were assessed according to the Deauville scoring criteria, named as Deauvilleinterim and Deauvilleend, respectively. The PET/CT data for patients with HT and without HT are presented in Table 3. Median SUV_baseline_ in patients with HT vs. without HT was 16.9 (range 2.12–29.72) vs. 7.9 (range 0.7–46.6), respectively; median SUV_interim_ was 2.49 (range 0–17.3) vs. 3.0 (range 0–11.4), respectively; and median SUV_end_ was 7.3 (range 0–20.2) vs. 2.5 (range 0–15.5), respectively. The proportion of patients with higher qualitative assessment Deauville_interim_ (scored 5–6 points) was 25% (2/8) and 10.9% (6/55) in the HT and non-HT groups, respectively; the percentage of patients classified as higher at Deauville_end_ (scored 5–6 points) was 50% (3/6) and 14.2% (10/70) in the HT and non-HT groups, respectively.Univariate Cox regression analysis showed that the SUV_baseline_ (HR = 1.073, 95%CI 1.031–1.115, P = 0.000) and SUV_end_ (HR = 1.163, 95%CI 1.023–1.323, P = 0.021) could predict HT. Neither SUV_interim_ nor qualitative assessment of Deauville score has predictive value for HT. A total of 128 patients with pathological grade 1–2 received multiple mild treatments and 91 patients with pathological grade 3a received rituximab-based systemic chemotherapy. To minimize the confounding effects of pathological classification and treatment strategies on the results, we conducted subgroup analysis in low (grade 1–2) and high (grade 3a) pathological grade patients, respectively. The result showed the baseline SUV_max_ was still an important predictor for HT in both high-grade (HR = 1.055, 95%CI 1.009–1.103, P = 0.017) and low-grade subgroups (HR = 1.224, 95%CI 1.033–1.449, P = 0.019); SUV_end_ also remained as a prognostic indicator for HT in high-grade subgroup (HR = 1.147, 95%CI 1.022–1.288, P = 0.020). Due to the insufficient sample size, statistical data on predictive value of SUV_end_ in low-grade subgroup could not be obtained. In addition, we conducted a multivariate Cox analysis of adjusted by FLIPI2 score on the significant indicators screened by univariate analysis, and found the SUV_baseline_ (HR = 1.065, 95%CI 1.020–1.111, P = 0.004) and SUV_end_ (HR = 1.261, 95%CI 1.076–1.478, P = 0.004) remained predictive factors of HT risk independently of FLIPI2 score.

**Table 3:** Univariate Cox proportional hazard regression analysis of PET/CT parameters in predicting HT risk

	HT	Non-HT	HR	95%CI	*P*-value
SUV_baseline_ (median, range)	16.9 (2.12–29.72)	7.9 (0.7–46.6)	1.073	1.031–1.115	0.000
Low-grade subgroup	9.64 (7.1–24.7)	7.7 (0.7–24.7)	1.224	1.033–1.449	0.019
High-grade subgroup	19.3 (2.12–29.72)	8 (1.5–46.6)	1.055	1.009–1.103	0.017
SUV_interim_ (median, range)	2.49 (0–17.3)	3 (0–11.4)	1.113	0.955–1.298	0.170
Low-grade subgroup	17.3 (17.3–17.3)	3.7 (0–11.4)	–	–	–
High-grade subgroup	2.6 (0–14.5)	2.5 (0–10.7)	1.078	0.861–1.351	0.512
SUV_end_ (median, range)	7.3 (0–20.2)	2.5 (0–15.5)	1.163	1.023–1.323	0.021
Low-grade subgroup	–	2.9 (0–15.5)	-	–	–
High-grade subgroup	8.5 (0–20.2)	0.57 (0–8.8)	1.147	1.022–1.288	0.020
Deauville_interim_ (1–3/4/5–6)	6/0/2	25/24/6	0.926	0.578–1.482	0.784
Deauville_end_ (1–3/4/5–6)	2/1/3	44/16/10	1.306	0.829–2.054	0.249

### Kaplan–Meier survival analysis

**Fig. 2:**
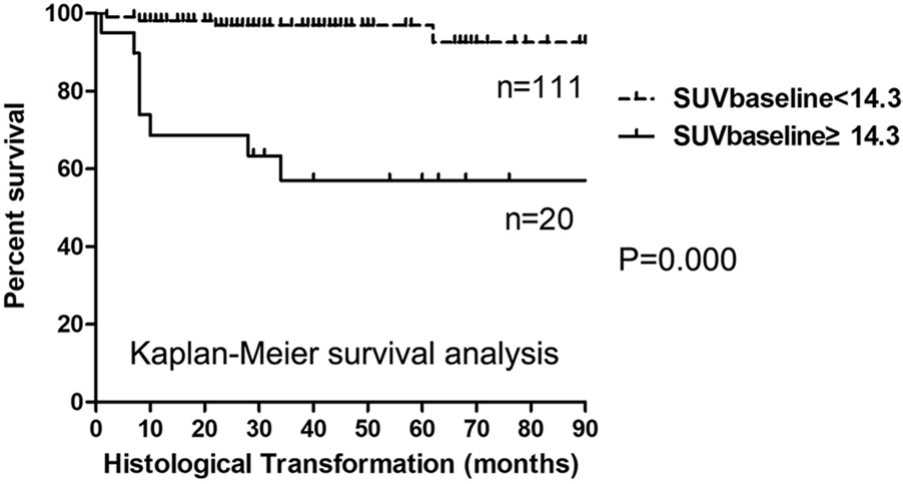
Kaplan–Meier survival analysis of time to HT in FL according to SUV_baseline_ value

**Fig. 3:**
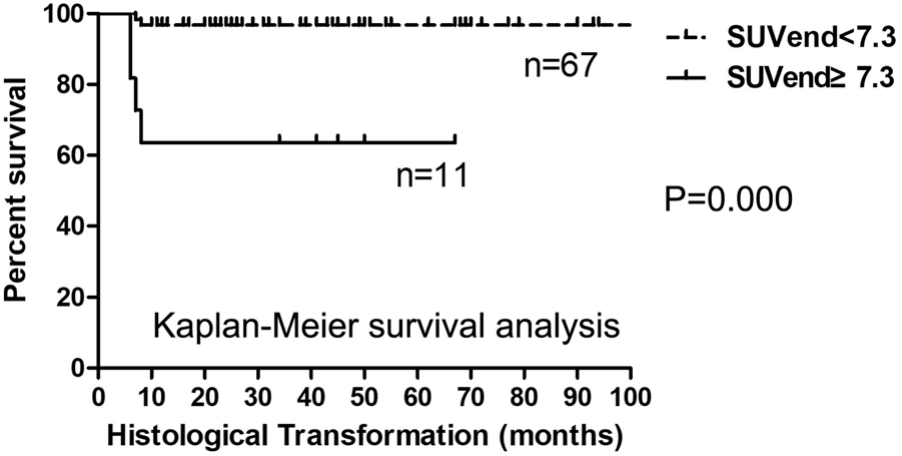
Kaplan–Meier survival analysis of time to HT in FL according to SUV_end_ value

We used ROC curve analysis to determine the accuracy and optimal cut-off values of SUV for predicting HT. The Area Under the Curve (AUC) for SUV_baseline_ and SUV_end_ was 0.761 and 0.669, respectively; their cut-off values were 14.3 and 7.3, respectively. A Kaplan–Meier survival analysis was performed according to the cut-off values. The median time to HT were 96 months and not reached between patients with SUV_baseline_ above and below the 14.3 cut-off, respectively. The higher SUV_baseline_ was significantly associated with a shorter time to HT (log-rank test, P = 0.000, Fig. 2). In addition, a significant difference in time to HT was also observed between patients with SUV_end_ above and below the 7.3 cut-off (logrank test, P = 0.000) (Fig. 3).

## Discussion

Follicular lymphoma shows a heterogeneous clinical course ranging from long-term responses to initial therapy to frequent recurrences or transformation to more aggressive subtypes. Histological transformation is a serious clinical event with a marked effect on the disease outcomes. In the past few decades, the incidence of HT has been reported to vary from 10 to 70% [2, 18–22]. This variability has been attributed to several factors, including the lack of a unique histological definition for transformation, the heterogeneity in study populations, differences in treatment strategies, and differences in the follow-up period. In our study, the rate of HT of FL was 13.6% over a median follow-up period of 46 months, which was slightly higher than the recently reported values. The HT incidence in high-grade patients was 26.4%, which was significantly higher than that in low-grade patients (4.7%). We speculate that a higher proportion of patients with advanced 3a pathological grade (more than 50%) may be a non-negligible reason for the increase in the HT rate in our study.

Even though in rituximab era, HT is still a serious event affecting the prognosis and survival of follicular lymphoma, especially in patients with subsequent transformation, with a 5-year PFS rate of less than 20% and 5-year OS rate of less than 70% [3]. The transformation of follicular lymphoma is a multi-step event, from an indolent lymphoma to a more aggressive lymphoma subtype, which is accompanied by an increase in tumor burden. Early screening to identify patients with high HT risk, and giving more aggressive treatment strategies may help reduce the occurrence of transformation, thereby reduce serious complications caused by high tumor burden [23] and improve patient survival. In 2018, Federico et al. conducted a retrospective pooled analysis to explore the risk factors influencing FL transformation [7]. In their study, 8116 patients from 11 cooperative groups or institutions across Europe were eligible for analysis, and the 10-year cumulative hazard of HT was 7.7%, with a high FLIPI score, grade 3a follicular lymphoma, and a previous watch-and-wait approach were associated with increased cumulative hazards of HT. In addition, in the PRIMA study, the risk factors for HT were high LDH levels, an altered performance status, anemia and systemic symptoms [24]. This has been well documented for most of the individual clinical to confirm the adverse factors, such as elevated serum LDH, high values of ECOG-PS, IPI and FLIPI2, but these indicators have no strong power to predict HT risk with weaker HR values ranging from 1 to 2 [3–6]. We speculate most transformations in FL are from the divergent evolution of small progenitor clones that is usually not detectable at diagnosis, which makes it difficult to predict it reliably with available clinical indicators. In this study, we also identified higher FLIPI2 score and 3a pathological grading were independent predictors of HT. Advanced pathological grade was shown to be most significantly related to HT risk with HR value > 4, while FLIPI2 showed a weaker HR value of only 1.55. Notably, the coagulation factor fibrinogen was a newly identified possible important factor predicting transformation. At present, research on the clinical significance of fibrinogen in FL is limited. Elevated pretreatment levels of fibrinogen are reported to be unfavorable predictors in clinical response and prognosis in DLBCL [25] and nasal-type natural/killer T cell lymphoma [26]. For FL, one study reported that elevated CRP levels showed a significant association with elevated fibrinogen levels, and patients with higher CRP levels had a significantly shorter PFS, implicating the potential poor prognosis of fibrinogen in FL [27]. Treatment strategies on the risk of HT in FL were also investigated. The largest study to date has shown that patients who are managed by watch and waiting are at increased HT risk [5]. A retrospective international study involving nearly 10,000 patients also stated that patients who did not receive rituximab as the initial treatment had an increased risk of HT [28].

Recently, some studies have investigated the potential predictive value of quantitative parameters of PET/CT for FL prognosis. As the most commonly used semi-quantitative indicator in PET/CT, SUV_max_ reflects the glucose metabolism of tumors, and has been proved to be closely related to tumor invasion [14, 29, 30]. Rossi et al. reported that baseline SUV_max_ > 14.5 was associated with poorer PFS than baseline SUV_max_ ≤ 14.5 [12]. With the development of software programs, TMTV may provide additional valuable information to assess response to treatment and patient prognosis. MTV is an indicator of the tumor score in vivo and may be a better estimator of the tumor burden than anatomical imaging. TMTV measured on the basis of baseline PET examinations can also predict PFS and overall survival (OS) better than the FLIPI score, thereby indicating progression within 2 years [13]. Cottereau et al. established a model including TMTV and PET_end_ and found that a high TMTV (> 510 cm^3^) and positive PET_end_ results were independent important risk factors for PFS [30]. In addition, for the qualitative evaluation of PET/CT, one study in a pooled analysis of three prospective trials demonstrated the prognostic value of Deauville score in FL. They found the positive results in post-induction PET scan (D5PS of 4, 5) could identify a high-risk group with shorter PFS, and indicated a significantly higher risk of death [14]. Boo et al. also retrospectively reviewed 33 FL patients who had three sets of initial staging, interim and end-of-therapy to evaluate the predictive value of Deauville score. The results confirmed that patients with interim and end-of –therapy D5PS of 4 or 5 showed shorter PFS than patients with scores 1, 2, or 3, and terminal Deauville score is an independent factor in FL progress [30].

In term of relationship between PET/CT and HT in FL, retrospective studies showed that the nodal sites of HT may have a higher SUV_max_ than non-transformed sites on PET/CT scans. Numerous studies have confirmed that higher SUV_max_ values increase the odds of higher grade or more aggressive lymphoma, and PET/CT is often used as an important non-invasive diagnostic tool to find the best biopsy site when transformation is suspected [16]. To date, only a few studies have assessed the ability of pre-transformation PET/CT parameters for predicting the risk of transformation in early stage of disease. A brief report published on Blood in 2020 assessed the relationship between baseline SUV_max_ and the risk for HT in FL from the GALLIUM study [17]. They found no temporal relationship between baseline SUV_max_ and HT. Neither baseline SUV_max_ nor baseline SUV_range_ could predict HT, suggesting that repeat biopsy of lesions may offer limited benefit in excluding HT based on SUV_max_ alone before initiating therapy in patients with FL with a high tumor burden. This study highlighted for the first time the insignificance of baseline SUV_max_ in predicting transformation risk, but the limited sample size (only 15 patients with HT) and a single population (patients with high tumor burden) may lead bias in results. Moreover, the usefulness of mid-term and terminal PET/CT assessments in predicting HT has not been reported to date. Therefore, we retrospectively collected a decade data to analyze the predictive power of pre-transformation PET/CT for the risk of HT in FL. All patients had no evidence of histological transformation when underwent PET/CT scans.

Our result showed that in quantitative assessments, higher levels of initial and post-treatment SUV_max_ both indicated a shorter time to HT. While in visual analysis, the results of the interim or post-treatment Deauville 5-point scale assessment, which uses liver activity as the reference, were not associated with the time to HT in FL. In our study, grade 3a disease was found to be the most important factor affecting transformation, and treatments containing rituximab have been reported to be closely related to a reduced incidence of transformation. Here, the treatment protocols for patients with grade 3a disease (R-CHOP or R-CVP) were more uniform than those for low-grade patients. To minimize the confounding effects of pathological classification and treatment strategy, we analyzed the predictive ability of PET/CT parameters for HT in low-grade and high-grade subgroups, respectively. The results showed the SUV _baseline_ was still an important predictor for HT in both high-grade and low-grade subgroups. SUV_end_ also remained as a prognostic indicator for HT in high-grade subgroup. These findings imply that a higher baseline SUV_max_ represents higher tumor invasiveness, leading to a higher probability of subsequent transformation, and poor efficacy of induction therapy is also an important factor influencing transformation. The NCCN-FLIPI2 was proposed recently and proved more accurate for identifying high-risk patients in the era of immune-chemotherapy. In our study, the NCCN-FLIPI2 score was also shown to be highly predictive for patients at risk of transformation. Therefore, we used multivariate analysis to evaluate the prognostic values adjusted by NCCN- FLIPI2 score, and concluded that SUV_baseline_ and SUV_end_ were significant HT predictors independent of NCCN- FLIPI2 score.

Recently, cytogenetics and tumor microenvironment to predict HT have also been studied. No single gene lesion could explain all cases of HT. TP53 mutation, loss of CKDN2A, and gains of MYC were reported to be participated in development of HT [31]. The microenvironment of patients with HT seems to be different but HT cannot be predicted based on microenvironment gene expression profile (GEP) [32, 33]. In addition, some emerging technologies such as the detection of blood ctDNA [16] and the application of Raman-Enhanced Spectroscopy (RESpect) probe to identify molecular chemical composition in tissue [34] may be used as potential diagnostic or predictive tools for the HT of FL.

Our study has several limitations. First, the study design was retrospective. Second, although the study included 219 patients, the PET/CT dataset provided was insufficient. The PET/CT data for the pre-treatment phase, interim period, and the end of treatment were obtained from scattered patients, and data for all three stages were available for very few individual patients. The lack of parallel data of patients and insufficient data for analysis reduced the reliability of the conclusion. In addition, although the initial biopsy-confirmed HT did not occur, sites with a high SUV_max_ may not be suitable for biopsy, and the possibility of de novo transformation cannot be completely ruled out.

## Conclusions

Although HT occurs in a minority of patients with follicular lymphoma, it significantly worsens the survival, prompting the clinical significance of early screening of high-risk HT patients. The biology of HT is complex, no single genetic lesion or mechanism could explain all cases of HT, and no clinical indicators or biomarks to strongly predict HT have been identified to date. PET/CT is used as a non-invasive diagnosis tool to help determine the best biopsy site when suspecting HT, but its role in predicting the HT risk in the early stage of disease is still unclear. The present study provides a decade of retrospective data to analyze the relationship between HT risk and PET/CT parameters at the initial, interim and end-treatment stages. SUV_baseline_ and SUV_end_ were validated to show predictive value on HT risk independently of FLIPI2 score, and may help to identify high risk patients in clinical. Given the bias caused by the analysis of scattered data in this study, more prospective studies with larger samples are needed to confirm the predictive ability of pre-trasformation PET/CT and whether patients could benefit from a change in treatment strategy based on PET/CT-based risk stratification of HT.

## Supplementary Information

**Additional file 1**. The original data.

## Data Availability

The datasets used and/or analysed during the current study are available from the corresponding author on reasonable request.

## Abbreviations

HT: Histological transformation
SUVmax: Maximum standardized uptake value;
PET: Positron emission tomography
18F-FDG PET/CT: 18F-fluorodeoxyglucose positron emission tomography/computed tomography
PETinterim: Interim PET
PETend: End-of-induction therapy
FLIPI2: FL International Prognostic Index 2
PETbaseline: PET at baseline
FL: Follicular lymphoma
NHL: Non-Hodgkin's lymphoma
DLBCL: Diffuse large B-cell lymphoma
D5PS: Deauville standard comprising a 5-point scale
HGB: Hemoglobin
HRs: Hazard ratios
CI: Confidence intervals
ROC: Receiver operating characteristic
GELF: Groupe d’Etude des Lymphomes Folliculaires
ISRT: Involved site radiation therapy
AUC: Area Under the Curve
TMTV: The total metabolic tumor volume
OS: Overall survival
bSUVmax: Baseline SUVmax
